# A network meta-analysis of the timing of wound dressing removal

**DOI:** 10.1308/rcsann.2023.0083

**Published:** 2024-07-31

**Authors:** RJKS Hwang, DL Crook, CS Allan, S Sarkar

**Affiliations:** Barts Health NHS Trust, UK

**Keywords:** wound dressing, wound infections, postoperative infections, dressing removal

## Abstract

**Introduction:**

Wounds are invariably dressed postoperatively but the evidence for the timing of dressing changes is limited. This meta-analysis evaluated whether the relative risk of wound infection varies depending on when dressings are changed.

**Methods:**

A frequentist random-effects network meta-analysis was conducted on the results of a systematic review of the MEDLINE^®^, Ovid^®^, Scopus^®^, Web of Science™ and PubMed^®^ databases and the Cochrane Central Register of Controlled Trials performed in May 2023. Evidence quality was graded using the Confidence In Network Meta-Analysis tool.

**Results:**

A total of 4 studies were included with 878 patients. A significant increase in the relative risk of wound infection was found when dressings were left in situ for more than 4.5 days when compared with 48 hours (3.18, 95% confidence interval: 1.22–8.33). There were no significant differences in the relative risk of infection between the other groups. Model heterogeneity and inconsistency were insignificant (Cochran's Q: 0.44, *p*=0.51). The quality of the evidence was graded as generally very low and risk of bias evaluations showed it to be of high concern for bias.

**Conclusions:**

Late dressing changes significantly increase the risks of wound infection and changes at 48 hours minimise these risks. There was no advantage demonstrated for earlier dressing changes. Ensuring that dressings are changed appropriately can minimise patient harm and health service costs.

## Introduction

### The condition

Surgical wounds facilitate access to underlying tissues and organs during surgical procedures, and are made and managed to optimise healing, minimise complications and promote recovery.^1^ After the surgical procedure is completed, the wound is closed using various techniques including sutures, staples or adhesive strips to ensure proper wound healing and reduce risk of infection.^2^

Wound healing is influenced by type and severity of the wound, including its size and depth. Adequate blood supply to the wound delivers oxygen, nutrients and immune cells necessary for healing. Infection impedes healing and increases the risk of complications. Chronic conditions such as diabetes, immune disorders and malnutrition can impair healing. Advanced age is often associated with a slower healing process. Certain medications, lifestyle factors like smoking and poor nutrition, chronic inflammation and mechanical factors such as excessive tension or pressure around the wound all affect wound healing.^3^ Conventionally, wounds and operations are classified as clean, clean-contaminated or contaminated^4^ and the characteristics of these are summarised in Table 1.

**Table 1:** Classification system for wounds and their characteristics^4^

Classification	Characteristics
Clean	No signs of inflammation. Does not involve repair or removal of an internal organ. Risk of infection <2%.
Clean-contaminated	No signs of infection at time of surgery but does involve removal of an internal organ. Risk of infection <10%.
Contaminated	Open fresh wounds and wounds that involve repair or removal of an internal organ. Bodily fluids spill from organ into wound. Risk of infection <20%.
Dirty-contaminated	Infection present at time of surgery. Risk of infection <40%.

The Centers for Disease Control and Prevention (CDC) define surgical site infection (SSI) as a post-surgical infection in the surgical site within 30 days of surgery and divide SSIs into three categories: superficial, deep or organ/space, depending on anatomical location.^5^ In January 2023, the CDC reported that SSIs are associated with a 2–11-fold increase in mortality rate, extending hospital admissions by 9.7 days and increasing the cost by $20,000 per admission.^5^ A meta-analysis from 2023 estimated the global incidence of SSIs to be 1.6–3.7%.^6^ Knowing the optimal timing for dressing removal could reduce the length of hospital admissions, improve resource allocation and enhance efficiency. Unnecessary and repeated dressing removal increases healthcare costs as well as burden on nursing staff, patient discomfort and disruption in the wound healing.^7,8^

### The intervention

The goals of dressing surgical wounds are to provide a protective barrier, maintain a moist environment and create an optimal environment for wound healing by minimising complications.^2^ Reducing the risk of bacteria entering the wound site prevents SSIs. The choice of dressing depends on various factors, including wound type and location, level of exudate and presence of infection.

The *British National Formulary* (*BNF*) provides general guidance on the selection and management of dressings.^9^ It emphasises maintaining cleanliness, using aseptic techniques during changes and ensuring appropriate wound bed preparation. The guideline highlights regular assessment for signs of infection, exudate and delayed healing. Transparent dressings allow visual inspection without re-dressing, enabling assessment of the wound's progress and prompt identification of signs of infection or other complications. Additionally, the *BNF* recommends using different dressings for specific wound characteristics (for example, adhesive films for low-exudate wounds, absorbent dressings for moderate to high-exudate wounds, hydrogels for dry or necrotic wounds and antimicrobial dressings for infected wounds). Moreover, certain dressings contain active substances or promote the release of growth factors to facilitate tissue regeneration and epithelialisation.^3^

Guidelines from individual NHS trusts advise that uncomplicated surgical wound dressings should be removed after at least 48 hours.^10,11^ However, the evidence base behind this remains unclear.^12,13^ Identifying the appropriate duration for dressing retention can inform optimal wound care guidelines to promote comfort and utilise resources efficiently.

## Methods

### Criteria for study selection

This systematic review and study was registered on the PROSPERO database (CRD42023421416) and has been conducted in line with the 2020 PRISMA (Preferred Reporting Items for Systematic reviews and Meta-Analyses) guidelines.^14^ Clinical trials were incorporated into our study without imposing restrictions based on blinding, randomisation, publication status, date of publication, study setting or sample size. Grey literature was excluded, as were any results for which the full text was not in current publication.

The patient population comprised patients with a surgical wound from both elective and emergency procedures. Studies were included irrespective of whether the surgical wounds were clean, clean-contaminated or contaminated and irrespective of type of surgery. Studies of children (aged <18 years) were excluded. We included studies whose intervention was early versus delayed dressing removal regardless of removal time. This was later adjusted in our statistical analysis. We excluded studies that examined no post-surgical dressing versus delayed surgical dressing removal. The outcome measured was wound infection (albeit defined by the original authors of the studies). Not enough evidence was found to investigate the outcome of wound dehiscence as originally intended in the PROSPERO registration.

### Search methods

The following six databases were searched:

1. MEDLINE^®^ (searched 01/05/23–07/05/23)

2. Ovid^®^ (searched 01/05/23–07/05/23)

3. Scopus^®^ (searched 01/05/23–07/05/23)

4. Web of Science™ (searched 01/05/23–07/05/23)

5. PubMed^®^ (searched 01/05/23–07/05/23)

6. Cochrane Central Register of Controlled Trials – 2022, issue 11

**Figure 1:**
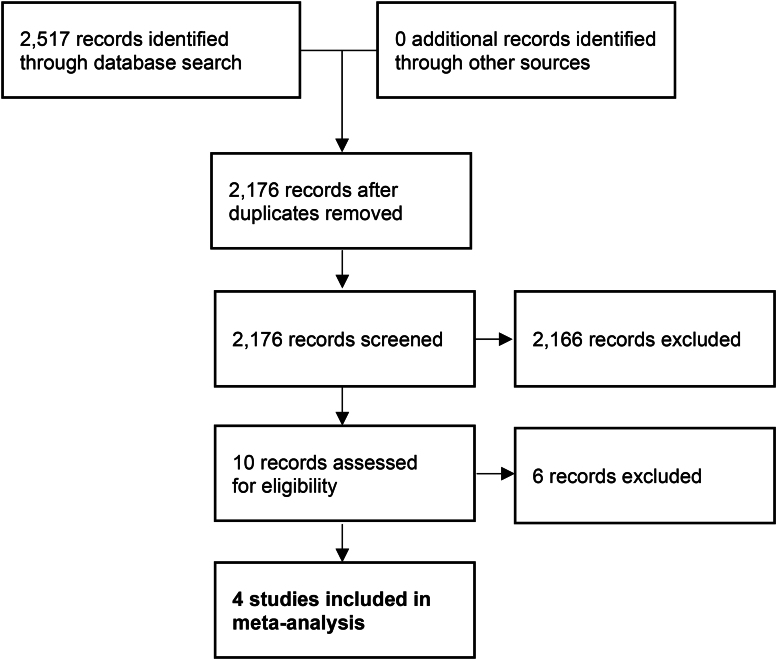
Flowchart of study selection

The search strategy was uniform for each database, using the MESH terms [MH bandages] and [MH surgical wound infection] and [MH surgical wounds] and [MH postoperative complications] as well as a free-text search of “early versus delayed removal”. Searches were not restricted by year of publication. The search strategy is outlined in Figure 1.

### Data collection and analysis

All data were extracted by two independent reviewers and any disagreements settled by the third. A frequentist random-effects network meta-analysis was conducted on the results of the systematic review using the netmeta package in R statistical software version 4.0.3 (R Foundation for Statistical Computing, Vienna, Austria; www.r-project.org).

In order to form a valid network for analysis and allow comparison between the papers, any timepoints of dressing change beyond 4.5 days were collapsed into a single group of >107 hours. Random-effects analysis was chosen owing to the likelihood that there is no single underlying true value to the results and owing to the anticipated heterogeneity. Heterogeneity and inconsistency were assessed using Cochran's Q test. P-scores, (frequentist simulations of SUCRA [Surface Under the Cumulative RAnking curve] values) for the relative superiority of each treatment were produced and appended to the forest plots of the model output. Sensitivity analysis was conducted through a fixed-effects meta-analysis of the data. Sources of inconsistency in the model design were sought using the inbuilt function netsplit of the netmeta package and through the use of network league plots. Traditional DerSimonian and Laird random-effects pairwise meta-analysis was also conducted of the available direct comparisons.

Cochrane Risk of Bias 2 (RoB 2) assessments were completed for the included studies^15^ and the quality of the evidence was assessed using the Confidence In Network Meta-Analysis (CINeMA) tool.^16^ Data and code are available on fair request to any of the listed authors.

## Results

There were 4 studies included in the network, comparing 878 patients, with 493 (56%) in an elective setting and 385 (44%) in an emergency setting).^17–20^ A total of 143 wound infections were noted across the papers (55 in patients who had elective surgery and 88 in patients who had emergency surgery). Of the 493 elective cases, 11.2% became infected; this compared with 22.9% of the 385 emergency cases.

The analysis compared four different times of dressing changes: at 48 hours, between 48 and 96 hours, at 96 hours and at >107 hours. The characteristics of these studies are summarised in Table 2, including the types of surgical procedures performed. No data were found on the degree of dressing soiling on removal or whether this information was used to form the assessment of wound infection. While there were inadequate data on degree of wound contamination to allow comparison between papers, the degree of wound contamination was reasonably homogenous (853 clean wounds [97.1%], 25 clean-contaminated wounds [2.9%]), with the study by Dosseh Eacute Koué *et al* being the only one to include clean-contaminated wounds.^17^ Lastly, most studies did not record the type of wound closure, which precluded this from analysis.

**Table 2:** Summary of the included studies

Study	Methods	Participants	Intervention	Outcomes	Type of surgery
Dosseh Eacute Koué, 2008^20^	RCT; 102 patients who underwent intra-abdominal surgery	Patients aged >18 years with clean or clean-contaminated wounds	Early dressing removal (48h) (*n*=51) vs late removal (>48h) (*n*=51); in the late group, dressing changes were performed every 48h	Postoperative temperature curve and incidence of SSIs	*Early group*: 28 herniorrhaphies, 13 thyroidectomies, 3 diagnostic laparotomies, 2 lipoma removals, 2 mastectomies, 1 pancreatectomy *Late group*: 19 herniorrhaphies, 5 thyroidectomies, 1 each of diagnostic laparotomy, lipoma removal, mastectomy, colostomy reversal, gastric ulcer repair, feeding jejunostomy and hysterectomy
Hafez, 2016^18^	RCT; 70 patients who underwent open surgical procedures	Patients aged 18–65 years with clean surgical wounds and controlled chronic diseases	Early dressing removal (48h) (*n*=35) vs late removal (120h) (*n*=35); wound care was performed in all patients every 48h	Incidence of SSI measured by appearance of erythema, hotness, swelling, pain, purulent discharge and separation at the surgical site	*Early group*: 4 thyroidectomies, 19 mastectomies, 9 herniorrhaphies, 2 cholecystectomies * Late group*: 8 thyroidectomies, 10 mastectomies, 13 herniorrhaphies, 4 cholecystectomies
Singh, 2022^19^	Open-label parallel-group randomised controlled study; 206 patients who underwent emergency and elective Caesarean sections	Patients aged 18–44 years with uncomplicated pregnancies and BMI <35kg/m^2^	Early dressing removal (48h) (*n*=103) vs late removal (120h) (*n*=103)	Primary outcome was to compare incidence of wound complications including SSIs	*Early group*: 103 emergency Caesareans *Late group*: 103 emergency Caesareans
Wadhwa, 2021^17^	RCT; 500 patients who underwent emergency and elective Caesarean sections	Patients aged >18 years; groups were comparable in terms of emergency vs elective setting, BMI and age	Early dressing removal (96h) (*n*=250) vs late removal (192h) (*n*=250)	Primary outcomes were measured using ASEPSIS scoring method	*Early group*: 50 elective Caesareans, 200 emergency Caesareans *Late group*: 70 elective Caesareans, 180 emergency Caesareans
BMI = body mass index; RCT = randomised controlled trial; SSI = surgical site infection					

**Figure 2:**
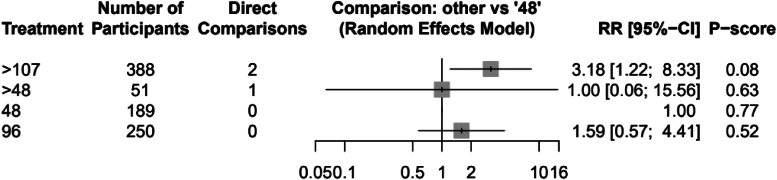
The forest plot of the network meta-analysis with P-scores for the mean extent of certainty of the superiority of each treatment in the network

Using dressing changes at 48 hours as the comparator, the RR of infection was found to be 1.00 (95% confidence interval [CI]: 0.06–15.56) for changes at 48–96 hours, 1.59 (95% CI: 0.57–4.41) for changes at 96 hours and 3.18 (95% CI: 1.22–8.33) for changes at >107 hours after surgery. P-scores (a measurement of the mean extent of certainty of the superiority of a treatment over the others in the analysis) suggested that dressing changes at 48 hours were superior to any other timepoint (P-score=0.77). The forest plot of the network meta-analysis is available in Figure 2. A standard network league table was produced of the findings (Table 3). This shows evidence from direct comparisons in the upper triangle and evidence from indirect comparisons in the lower triangle. There was agreement between the direct and indirect evidence, indicating consistency in the network.

**Table 3:** The network league table investigating the relative risks (with 95% confidence intervals) for different times of dressing changes, with evidence from direct comparisons in the upper triangle and from indirect comparisons in the lower triangle. Relative risks lower than 1 favour the column-defining intervention for the network meta-analysis results (lower triangle) and the row-defining intervention for the pairwise meta-analysis results (upper triangle)

**Pairwise meta-analysis (direct comparisons)**			
**>107 hours**		3.18 (1.22–8.33)	2.00 (1.43–2.80)
3.18 (0.17–58.32)	**>48 hours**	1.00 (0.06–15.56)	
3.18 (1.22–8.33)	1.00 (0.06–15.56)	**48 hours**	
2.00 (1.43–2.80)	0.63 (0.03–11.74)	0.63 (0.23–1.74)	**96 hours**
**Network meta-analysis (indirect comparisons)**			

**Figure 3:**
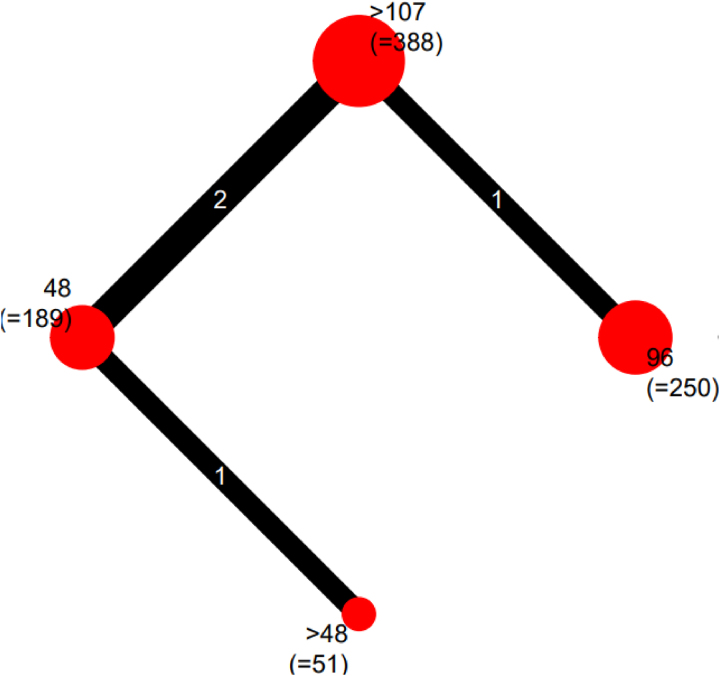
The network diagram of the relationships between the studies. The size of the node reflects the number of patients and the thickness of the line reflects the number of comparisons between nodes.

**Figure 4:**
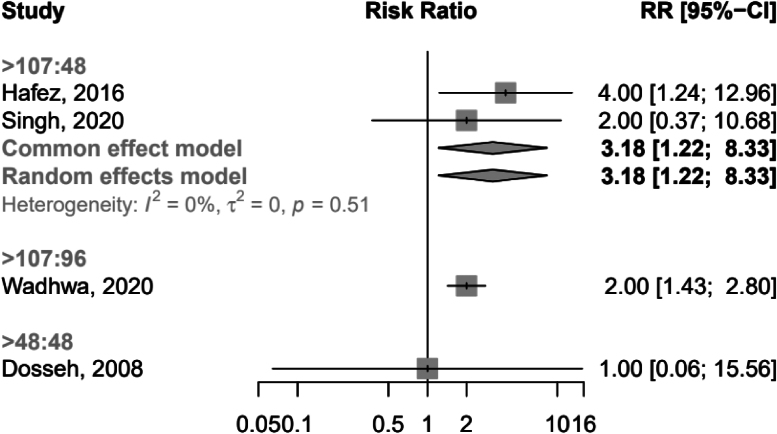
The forest plot of the pairwise comparisons in the meta-analysis with the relative risk of infection for each study where no pairwise comparisons were possible

**Figure 5:**
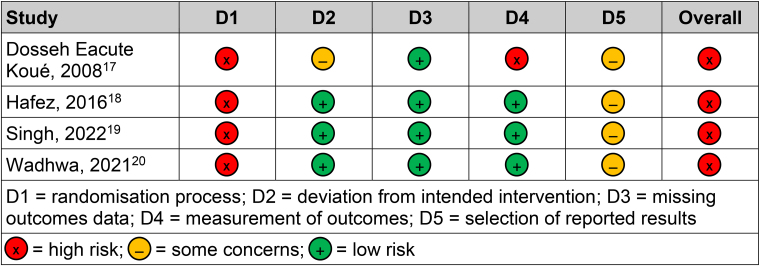
The output from the Cochrane Risk of Bias 2 tool^15^

**Figure 6:**
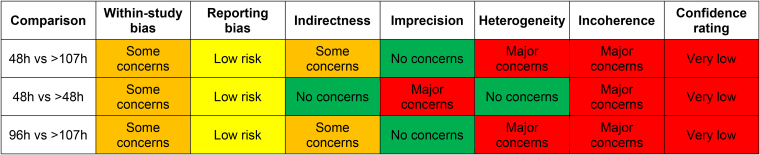
The output from the Confidence In Network Meta-Analysis tool^16^

The network diagram is shown in Figure 3. Heterogeneity and inconsistency in the model were insignificant (Cochran's Q: 0.44, *p*=0.51). The sensitivity analysis suggested that the modelling was robust, with no differences between the fixed and random-effects models. Traditional pairwise meta-analysis of the direct comparisons in the dataset showed an increased RR of infection when dressings were left for longer than 48 hours, with a pooled RR from the random-effects model of 3.18 (95% CI: 1.22–8.33). The forest plot for pairwise meta-analysis is available in Figure 4. This is consistent with the results of the network meta-analysis and indicates coherence in the findings of the model. The RoB 2 assessments demonstrated that the studies forming the network all had a high risk of bias (Figure 5) and accordingly, the CINeMA grading of confidence in the evidence was very low (Figure 6).

## Discussion

The results of this network meta-analysis suggest that dressings are best changed at 48 hours following surgery, with an increased risk of infection in wounds that have dressings left in situ for longer than this. Regular dressing changes remove debris that accumulates in the postoperative period, allow wound cleaning and reduce the bioburden of bacteria, which gather in the closed environment between wound and dressing. Furthermore, this relatively anoxic environment could encourage preferential growth of anaerobic pathogens in place of skin commensals. This can be seen as a mechanism via which dressing changes can reduce the risks of infection, and therefore patient harm and system cost.

There was no advantage observed for earlier dressing changes, which may be due to the small number of patients forming this group among the studies. However, it may equally feflect that, on average, dressings are less soiled at this timepoint and so bacterial growth among dressing debris has not reached a critical burden.

These findings are based on reasonably small studies, which contributed to the generation of the wide confidence intervals around the point estimates. In addition, the size and quality of these trials led to the evidence being graded as at risk of bias and there being low confidence in the outcomes. Many of the trials gave cause for concern with unclear processes for randomisation. None of the included trials were blinded in any way. The assessment criteria for wound infection varied between each study, giving the possibility of variation in the classification of the outcome. The estimates of the RRs are highly imprecise owing to the small size of the studies included and there is likely to be heterogeneity not accounted for in the analysis from the different methods and surgical sites in each study.

Greater reliability as to the size of the effect could be gained through a suitably powered randomised controlled trial (RCT) examining the timing of dressing removal, which could then be included in a meta-analysis and would also allow generation of primary evidence on dressing use against other strategies, such as use of tissue adhesives or no dressing, a question pertinent to current wound management research.^21^ While important for a comparative analysis to be made, it is essential to first optimise each strategy through several large RCTs. Unified techniques for aseptic dressing changes and classification of wound infections would need to be employed, and this could make coordination of such an RCT difficult and resource intensive. Finally, undertaking network meta-analyses in surgery is not widespread and the use of one here demonstrates the utility of the technique for producing more precise estimates of effect through collating relevant studies.

## Conclusions

This network meta-analysis found that changing the wound dressing at 48 hours after surgery was the best option of those examined. Later changes (beyond 4.5 days) increased the relative risk of infection. The studies included in this analysis were mostly small and at high risk of bias, and the CINeMA grading of confidence in the evidence was very low. However, within the limitations of the evidence available, it appears that 48 hours may be the best time to change abdominal wound dressings postoperatively.
